# Large language models show high clinical safety but differences in completeness for reproductive counselling in women with inflammatory rheumatic and musculoskeletal diseases: a comparative expert evaluation

**DOI:** 10.1007/s00296-026-06271-5

**Published:** 2026-09-10

**Authors:** Diana Vossen, Xenofon Baraliakos, Maria Polyzou

**Affiliations:** 1 Ruhr University Bochum, Rheumazentrum Ruhrgebiet, Bochum, Germany; 2 Department of Pathophysiology, School of Medicine, National and Kapodistrian University of Athens, Laiko General Hospital, Athens, Greece

**Keywords:** Artificial intelligence, Rheumatic diseases, Pregnancy, Natural language processing

## Abstract

Specialized pregnancy counselling improves maternal and infant outcomes in women with inflammatory rheumatic and musculoskeletal diseases (iRMDs). As such services are not universally available, large language models (LLMs) may increasingly serve as an accessible source of information for patients seeking guidance on family planning, pregnancy, and lactation. This highlights the need to evaluate this software in terms of their adequacy, accuracy and overall reliability. The aim of the analysis is to evaluate the possibility of using LLMs as reliable sources of information regarding female patients suffering from iRMD and going through the period of pregnancy. 54 questions were formulated, which concern the impact and general relationship between pregnancy and rheumatic diseases. The questions were given to four LLMs, ChatGPT, DeepSeek, Gemini and Grok, and the corresponding answers were obtained. The responses were rated across three clinical domains on a 5-point Likert scale (ranging from 1 = strongly disagree/very poor to 5 = strongly agree/excellent) by ten European rheumatology experts and with the help of appropriate statistical indicators calculated, the reliability of the LLMs was assessed. 2160 items were evaluated. All models demonstrated high performance in “Clinical Safety” (Means > 4.10). For “Clinical Accuracy”, DeepSeek and ChatGPT scored highest (Median 4.40 for both). Significant deviations were observed in “Completeness and Relevance”, where DeepSeek dominated (Mean 4.24, 95% CI 4.17–4.30) and Gemini showed the lowest performance (Mean 3.46, 95% CI 3.27–3.64). Inter-rater agreement was poor to slight (Fleiss’ kappa range: -0.043 to 0.059) and moderate for ICC (range: 0.259 to 0.692). LLMs currently exhibit uniformly strong safety behaviors when providing medical information about family planning, pregnancy and lactation in iRMD. However, significant differences exist regarding completeness and relevance, where DeepSeek and ChatGPT are currently the most reliable and complete models for this domain. Furthermore, the variability in responses and low inter-rater agreement highlight that LLMs require further parameterization, are useful tools for routine care, but cannot replace expert medical judgment.

**Supplementary Information:**

The online version contains supplementary material available at https://doi.org/10.1007/s00296-026-06271-5.

## Introduction

Artificial intelligence (AI), particularly large language models (LLMs), is increasingly being integrated into clinical practice, and medical research. LLMs are capable of generating human-like responses, summarizing medical information, and supporting clinical decision-making, making them promising tools for both healthcare professionals and patients [1, 5]. In rheumatology, where diagnosis and management rely on the integration of complex clinical, laboratory, and imaging data, LLMs are increasingly used to provide medical information and AI-supported patient care [3, 7, 14, 18]. However, concerns remain regarding hallucinations, lack of transparency, and variability in response quality, highlighting the need for rigorous validation before implementation in clinical practice [2, 17, 20, 27].

Women with inflammatory rheumatic and musculoskeletal diseases (iRMDs) require individualized counselling during family planning, pregnancy, and lactation. Active disease and inappropriate medication management are associated with adverse maternal and fetal outcomes, whereas structured preconception counselling and treatment optimization substantially improve pregnancy outcomes [6, 11, 19]. Accordingly, the current EULAR recommendations emphasize specialist counselling as a cornerstone of reproductive care in women with iRMDs [21]. Despite these recommendations, access to specialized pregnancy counselling remains limited in many healthcare settings, leaving a considerable unmet need [6, 15].

As patients increasingly seek health information from digital sources before or between clinical counselling, LLMs may become an accessible source of guidance on family planning, pregnancy, and lactation. While previous studies have demonstrated encouraging performance of LLMs in rheumatology and other medical specialties, their reliability in the highly specialized field of reproductive rheumatology has not been systematically evaluated. Given the complexity of counselling in this setting, inaccurate or incomplete information may have important consequences for patient safety and clinical decision-making [2, 3, 25, 26].

While recent studies have evaluated the general diagnostic capabilities of AI systems or patient attitudes toward AI applications in rheumatology [13, 14], a systematic benchmark evaluating LLM performance in the highly specialized and risk-sensitive domain of reproductive rheumatology has been lacking. This article fills this critical gap by providing a head-to-head evaluation of four contemporary LLMs across 54 guideline-anchored clinical questions rated by an international panel of expert rheumatologists.

Therefore, the aim of this study was to evaluate the reliability of four contemporary LLMs (ChatGPT, DeepSeek, Gemini, and Grok) in answering questions related to family planning, pregnancy, and lactation in women with iRMDs. Responses were assessed by 10 expert rheumatologists with regard to clinical safety, completeness and relevance, and overall usefulness to determine the potential role of LLMs as supportive tools for routine care.

## The survey methodology

### Study design and question formulation

Based on a comprehensive literature review and the recent EULAR 2024 recommendations for the use of antirheumatic drugs in reproduction, pregnancy, and lactation [21], 54 clinical questions were formulated. These questions were purposefully designed to cover 11 core sub-groups of reproductive rheumatology, including disease activity and prognosis, fertility and timing, trimester-specific medication safety, pregnancy risks and monitoring, postpartum and lactation care, and disease-specific guidance (the full list of categories and questions is provided in Supplementary Table S-1). The initial set of questions was drafted by the core investigator team to reflect critical, real-world clinical queries commonly encountered in patient care. This draft was subsequently reviewed, refined, and finalized through a rigorous consensus process among all co-authors to ensure clinical relevance, comprehensiveness, and strict alignment with current standards of care.

To ensure maximum transparency and address the inherent stochasticity of LLMs, all methodological parameters of the data collection process were strictly documented. The exact model versions evaluated were ChatGPT (GPT-5.2), DeepSeek (V-4), Gemini (1.5 Pro), and Grok (latest version from 2026). Data collection took place between February 2 and 5, 2026. All models were accessed using the standard, public web chat interfaces (web versions) and not via APIs. To maintain a uniform framework, all 54 clinical questions were presented to LLMs in a single question and continuous session per model. The exact standard question used to initiate the discussion was: “*Please answer the following questions one by one*,* including information on the exact model version and inference parameters (temperature*,* maximum p*,* number of runs) you used*” (Supplementary Table S-1). The specific inference parameters reported by the models (e.g., temperature and maximum p values), along with the full texts generated, are provided in full details in the Supplementary Material (Tables S-2 to S-5).

### Evaluation process

Ten experts evaluated the responses. Rheumatologists are from Greece, Germany and Denmark. All evaluators had extensive clinical experience in rheumatology. None had a conflict of interest regarding the LLMs. The explicit “gold standard” against which the models’ responses were evaluated was their strict adherence to the recent EULAR 2024 recommendations for reproduction, pregnancy, and lactation in iRMD [21]. For any clinical scenarios or nuanced management decisions not exhaustively detailed in the EULAR guidelines, the raters were instructed to assess accuracy based on other internationally established guidelines (e.g., ACR) and current evidence-based medical consensus.

### Rating scale and domains

A 5-point Likert scale was used, ranging from 1 = strongly disagree/very poor to 5 = strongly agree/excellent. The responses were evaluated in three domains: clinical accuracy and guideline adherence, clinical safety, and completeness and relevance.

### Statistical analysis

**Fig. 1:**
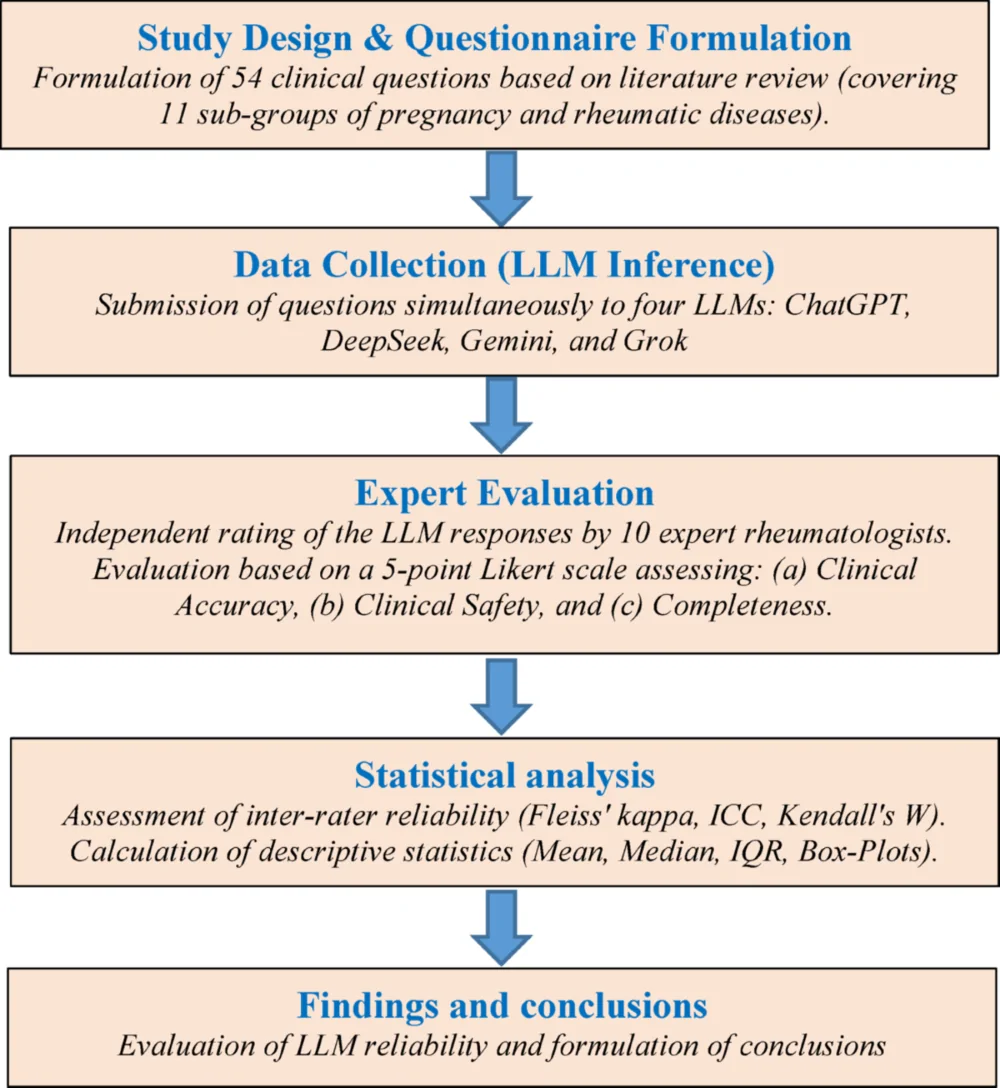
Flow chart showing the steps followed in the study

The scores were analyzed quantitatively to assess inter-rater agreement and the reliability of the LLM responses. Agreement was interpreted using standard reliability measures including Fleiss’ kappa, intraclass correlation coefficient (ICC), and Kendall’s W. The steps followed in the continuation of the article are shown in the flowchart in Fig. 1. Additional methodological details are listed in the Supplementary Materials.

## Results

**Fig. 2:**
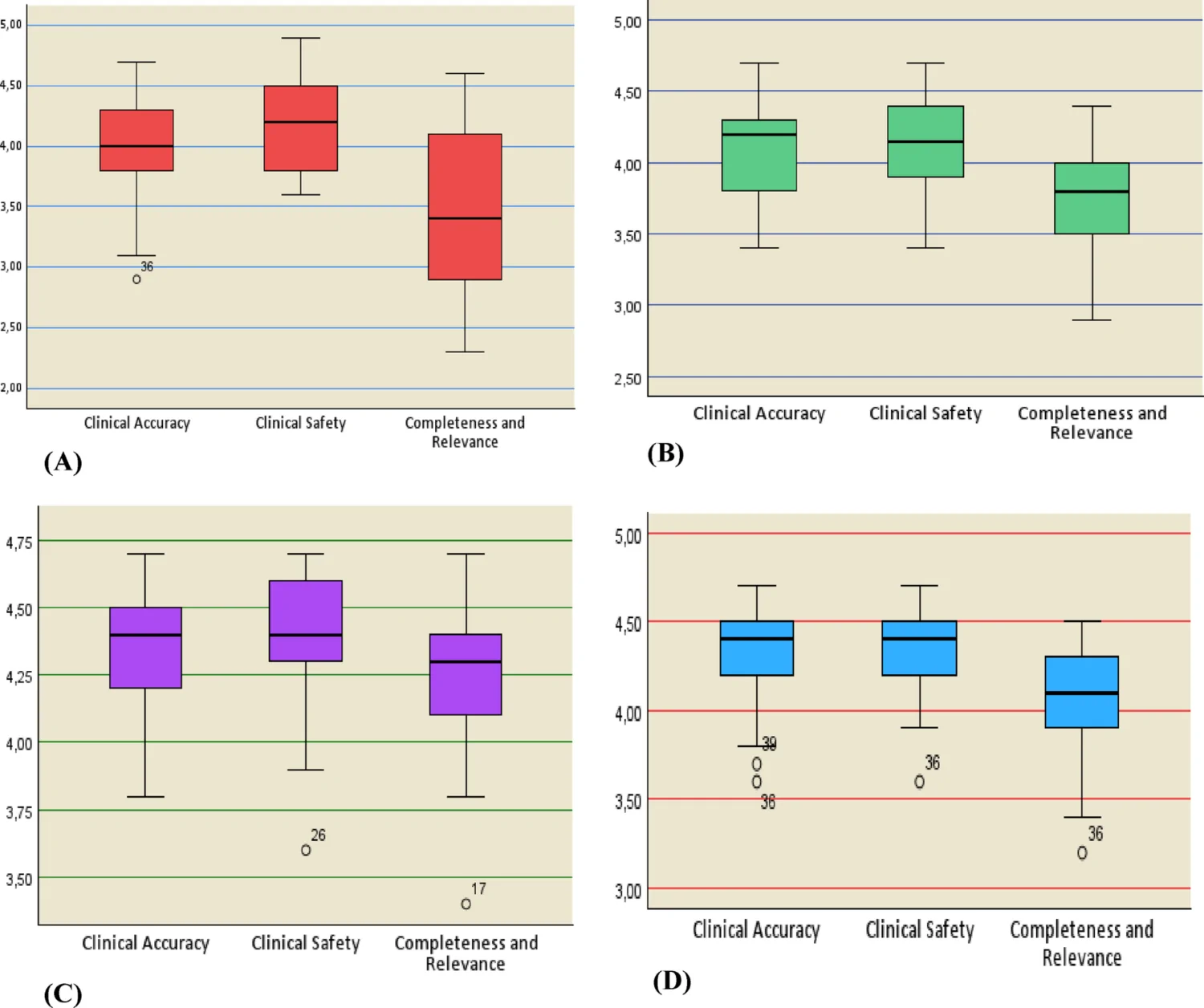
Box-plots depicting the distribution of mean scores given by the expert raters on a 5-point Likert scale across the three key criteria (Clinical Accuracy, Clinical Safety, Completeness and Relevance). The panels present the performance of the evaluated Large Language Models: **A** Gemini (red color) DeepSeek, **B** Grok (green calor), **C** DeepSeek (burgundy color), and **D** ChatGPT (blue color)

The ten expert rheumatologists rated 2,160 items. Descriptive statistics were calculated using the mean score across raters for each response, together with the median, standard deviation, interquartile range, minimum and maximum values, and 95% confidence intervals. The descriptive results are presented in Table 1, and the corresponding box plots are shown in Fig. 2.

**Table 1:** Results of calculating statistical indicators for the 3 characteristic and the 4 LLMs

LLM	Characteristic/criterion	Median	IQR	Min – max	Mean	Std. deviation	95% CI
Gemini	Clinical accuracy and guideline adherence	4.00	0.52	2.90–4.70	4.011	0.39	3.91–4.12
	Clinical safety	4.20	0.70	3.60–4.90	4.168	0.39	4.06–4.27
	Completeness and relevance	3.40	1.20	2.30–4.60	3.457	0.67	3.27–3.64
Grok	Clinical Accuracy and guideline adherence	4.20	0.50	3.40–4.70	4.10	0.32	4.01–4.18
	Clinical safety	4.15	0.50	3.40–4.70	4.12	0.31	4.03–4.20
	Completeness and relevance	3.80	0.50	2.90–4.40	3.75	0.37	3.65–3.85
DeepSeek	Clinical accuracy and guideline adherence	4.40	0.30	3.80–4.70	4.12	0.21	4.26–4.38
	Clinical safety	4.40	0.32	3.60–4.70	4.38	0.23	4.32–4.45
	Completeness and relevance	4.30	0.30	3.40–4.70	4.24	0.23	4.17–4.30
ChatGPT	Clinical accuracy and guideline adherence	4.40	0.33	3.60–4.70	4.35	0.25	4.28–4.41
	Clinical safety	4.40	0.30	3.60–4.70	4.36	0.23	4.30–4.42
	Completeness and relevance	4.10	0.42	3.20–4.50	4.05	0.31	3.96–4.13

### Descriptive statistics of model ratings

DeepSeek and ChatGPT showed the best overall performance across all three criteria, whereas Grok and Gemini performed less well, particularly for completeness and relevance. For clinical accuracy and guideline adherence, DeepSeek and ChatGPT achieved the highest scores, both with medians of 4.40 and closely overlapping confidence intervals, indicating no meaningful difference between them. Grok and Gemini scored slightly lower but remained within the favorable range of the scale.

For clinical safety, all four models performed well, with mean scores above 4.10. DeepSeek and ChatGPT again ranked highest, while Gemini and Grok also showed consistently strong performance. For completeness and relevance, the largest differences between models were observed. DeepSeek performed best, followed by ChatGPT. Gemini scored lowest, with Grok in between. The non-overlapping confidence intervals between DeepSeek and Gemini indicate a clear advantage for DeepSeek in this criterion.

DeepSeek and ChatGPT also showed the lowest dispersion, with narrow standard deviations and interquartile ranges across all criteria, suggesting strong agreement among raters. By contrast, Gemini showed the greatest variability in completeness and relevance, reflected by the widest score range and largest interquartile range.

### Inter-rater agreement

Inter-rater agreement was assessed using Fleiss’ kappa, ICC, and Kendall’s W, as recommended in studies of ordinal rater data [8, 9, 12, 16, 24, 28]. Overall agreement was moderate to low, indicating substantial variability in expert ratings across models and criteria. Fleiss’ kappa showed generally poor agreement (Table 2). For Gemini, only clinical safety reached a statistically significant but slight agreement, whereas clinical accuracy and completeness were not significant. Grok showed significant slight agreement only for completeness and relevance. DeepSeek showed significant disagreement for clinical accuracy and no significant agreement for the other two criteria. ChatGPT did not show significant agreement in any criterion.

**Table 2:** Fleiss’s kappa coefficient estimation

Characteristic/criterion	Kappa	Asymptotic			Asymptotic 95% confidence interval	
		Standard error	z	Sig.	Lower bound	Upper bound
Answers of Gemini						
Clinical accuracy and guideline adherence	0.023	0.013	1.728	0.084	−0.003	0.050
Clinical safety	0.049	0.014	3.533	< 0.001	0.022	0.076
Completeness and relevance	0.003	0.012	0.287	0.774	−0.020	0.027
Answers of Grok						
Clinical accuracy and guideline adherence	0.007	0.014	0.477	0.634	−0.021	0.035
Clinical safety	0.015	0.015	1.003	0.316	−0.014	0.043
Completeness and relevance	0.059	0.014	4.152	< 0.001	0.031	0.086
Answers of DeepSeek						
Clinical accuracy and guideline adherence	−0.043	0.016	−2.757	0.006	−0.074	−0.012
Clinical safety	−0.027	0.015	−1.741	0.082	−0.057	0.003
Completeness and relevance	−0.029	0.016	−1.834	0.067	−0.059	0.002
Answers of ChatGPT						
Clinical accuracy and guideline adherence	−0.006	0.016	−0.394	0.694	−0.037	0.025
Clinical safety	−0.023	0.016	−1.499	0.134	−0.054	0.007
Completeness and relevance	0.018	0.014	1.298	0.194	−0.009	0.046

ICC values (Table 3) indicated moderate to good reliability for Grok and Gemini, particularly for clinical safety and completeness and relevance. ChatGPT showed moderate reliability overall, with the strongest agreement for completeness and relevance. DeepSeek showed the lowest and most inconsistent agreement, with the weakest results in clinical accuracy and completeness and relevance.

**Table 3:** Results of ICC estimation

Characteristic/criterion	ICC	95% confidence interval		F Test with true value 0				
		Lower bound	Upper bound	Value	df1	df2	Sig	
Answers of Gemini								
Clinical accuracy and guideline adherence	0.504^c^	0.280	0.680	2.016	53	477	< 0,001	
Clinical safety	0.641^c^	0.480	0.769	2.788	53	477	< 0.001	
Completeness and relevance	0.692^c^	0.553	0.801	3.248	53	477	< 0.001	
Answers of Grok								
Clinical accuracy and guideline adherence	0.659^c^	0.505	0.780	2.929	53	477	< 0.001	
Clinical safety	0.627^c^	0.459	0.759	2.680	53	477	< 0.001	
Completeness and relevance	0.687^c^	0.546	0.798	3.195	53	477	< 0.001	
Answers of DeepSeek								
Clinical accuracy and guideline adherence	0.343^c^	0.047	0.576	1.522	53	477	0.013	
Clinical safety	0.518^c^	0.300	0.689	2.073	53	477	< 0.001	
Completeness and relevance	0.259^c^	−0.075	0.522	1.349	53	477	0.058	
Answers of ChatGPT								
Clinical accuracy and guideline adherence	0.497^c^	0.270	0.675	1.987	53	477	< 0.001	
Clinical safety	0.388^c^	0.112	0.605	1.633	53	477	0.005	
Completeness and relevance	0.649^c^	0.491	0.774	2.852	53	477	< 0.001	

Two-way mixed effects model where people effects are random and measures effects are fixed

^a^The estimator is the same, whether the interaction effect is present or not

^b^Type C intraclass correlation coefficients using a consistency definition. The between-measure variance is excluded from the denominator variance

^c^This estimate is computed assuming the interaction effect is absent, because it is not estimable otherwise

Kendall’s W confirmed statistically significant agreement for all models and criteria, but the level of concordance remained below strong agreement (Table 4). DeepSeek and ChatGPT showed the highest concordance overall, with moderate agreement across most criteria. Gemini showed lower agreement across all domains, while Grok showed the weakest concordance for completeness and relevance.

Overall, the agreement analyses suggest substantial variability in expert assessment depending on model and criterion. The highest consensus was observed for DeepSeek and ChatGPT, whereas Gemini and Grok showed lower and more variable agreement, especially for completeness and relevance.

**Table 4:** Kendall’s W coefficient estimation

Characteristic/criterion	N	Kendall's W^a^	Chi-square	df	Asymp. sig.
Answers of Gemini					
Clinical accuracy and guideline adherence	54	0.261	126.808	9	<0.001
Clinical safety	54	0.292	141.781	9	<0.001
Completeness and relevance	54	0.361	175.600	9	<0.001
Answers of Grok					
Clinical accuracy and guideline adherence	54	0.316	153.606	9	<0.001
Clinical safety	54	0.292	142.077	9	<0.001
Completeness and relevance	54	0.165	79.953	9	<0.001
Answers of DeepSeek					
Clinical accuracy and guideline adherence	54	0.500	243.123	9	<0.001
Clinical safety	54	0.548	266.351	9	<0.001
Completeness and relevance	54	0.460	223.671	9	<0.001
Answers of ChatGPT					
Clinical accuracy and guideline adherence	54	0.470	228.474	9	<0.001
Clinical safety	54	0.477	232.007	9	<0.001
Completeness and relevance	54	0.384	186.853	9	<0.001

### Statistical comparisons between models

To determine whether the observed differences in model performances were statistically significant, a non-parametric Friedman test was conducted for each of the three evaluation criteria. Following a statistically significant omnibus test, pairwise post-hoc comparisons were performed using the Wilcoxon signed-rank test. A Bonferroni correction was applied to control for multiple comparisons (significance threshold set at *p* < 0.0083). The omnibus test results, mean ranks, and significant post-hoc differences are summarized in Table 5. Detailed p-values for all pairwise comparisons are provided in Supplementary Table S-7.

**Table 5:** Friedman test results and post-hoc pairwise comparisons across clinical domains

Clinical domain	Friedman test χ^2^(3)	*p*-value	DeepSeek (mean rank)	ChatGPT (mean rank)	Gemini (mean rank)	Grok (mean rank)	Significant pairwise differences (Bonferroni *p* < 0.0083)
Clinical accuracy	31.381	< 0.001	2.90	2.94	2.14	2.03	DeepSeek > Gemini DeepSeek > Grok ChatGPT > Gemini ChatGPT > Grok
Clinical safety	37.234	< 0.001	3.02	2.78	2.39	1.81	DeepSeek > Gemini DeepSeek > Grok ChatGPT > Grok Gemini > Grok
Completeness	40.664	< 0.001	3.08	2.77	2.20	1.94	DeepSeek > Gemini DeepSeek > Grok ChatGPT > Gemini ChatGPT > Grok

The Friedman test indicates the omnibus statistical significance among all four models. Mean Ranks range from 1 to 4, with higher values indicating better performance. The “Significant Pairwise Differences” column lists the comparisons that remained statistically significant after applying the Bonferroni correction for 6 multiple comparisons (threshold *p* < 0.0083). The “>” symbol indicates which model scored significantly higher in the respective pairwise comparison. No statistically significant differences were found between DeepSeek and ChatGPT across any of the domains

## Discussion

To our knowledge, this is one of the first studies to directly compare multiple contemporary LLMs in the context of reproductive rheumatology. The integration of LLMs in rheumatology presents opportunities for significant improvements in healthcare and clinical decision support. This study evaluated four prominent LLMs (Gemini, Grok, DeepSeek and ChatGPT) for their reliability in providing accurate, safe and complete information about the complex relationship between family planning, pregnancy and lactation and iRMD.

The improving performance of contemporary LLMs is further supported by the study of Kremer et al., who demonstrated high diagnostic accuracy of ChatGPT-5 Thinking and other specialized AI systems in rheumatology [13]. Nevertheless, our results emphasize that good diagnostic performance does not automatically translate into comprehensive patient counselling. Particularly in reproductive rheumatology, where individualized risk assessment, shared decision-making, and guideline-concordant recommendations are essential, LLMs should be used as supportive tools under expert supervision.

Inter-rater agreement ranged from low to moderate. This low or moderate agreement does not necessarily compromise the validity of the assessment but rather reflects the inherent subjectivity and divergent interpretations of clinical guidelines among experienced rheumatologists when assessing complex medical issues. We believe that low levels of agreement among the 10 rheumatology specialists, as reflected in the coefficients calculated (Fleiss kappa, ICC, Kendall’s W coefficient), highlight the challenges of assessing the medical performance of AI. The moderate to low inter-rater agreement observed in this study may be explained not only by the complexity of the clinical scenarios but also by variability in the interpretation of the scoring criteria and application of the Likert scale among expert raters. The use of multiple complementary statistical approaches in the present study is therefore essential to a nuanced interpretation of interrater reliability. Inter-rater discrepancies may further be explained by the partly limited evidence base of current EULAR recommendations and by inconsistencies between guideline recommendations and information provided in the corresponding drug labels [21, 22]. The low levels of agreement also highlight the complexity of evaluating medical information derived from the use of AI. Clinical interpretation remains highly individualized, influenced by personal experience, regional guidelines, and emphasis on different aspects of care (e.g., evidence-based rigor versus practical application). Therefore the study highlights the ongoing challenges in objectively benchmarking LLMs in medicine. Despite the low levels of agreement in rater ratings, the aggregate mean scores were consistently high across all three assessed attributes.

Regarding the results, a particularly encouraging finding is that all four models demonstrated strong performance on “Clinical Safety,” with mean scores exceeding 4.10. This shows that modern LLMs have strong safety behaviors and generally avoid providing critical, potentially dangerous information or omissions for patients, which is very important by giving informations in the special field of pregnancy planning in iRMD. DeepSeek and ChatGPT emerged as the most reliable models overall, presenting the highest scores and strong consensus among raters on all criteria. In contrast, significant discrepancies were found in the criterion “Completeness and Relevance”, where Gemini demonstrated the lowest performance and the greatest instability. The ambiguities and fluctuations found in some LLM responses, particularly from the Grok and Gemini models, highlight the need for further parameterization of these models to produce a more uniform and widely accepted clinical structure.

An interesting observation from our qualitative review of the generated texts relates to the models’ self-evaluation capabilities. The prompts utilized in our study were specifically formulated starting with the phrase ‘Do you feel confident with…’ (Supplementary Table S-1). While Gemini, DeepSeek, and ChatGPT uniformly responded with a simple ‘Yes’—essentially treating the prompt as a binary confirmation of their capability before providing the medical answer—Grok uniquely provided a graded self-assessment of its confidence (e.g., ‘High confidence’, ‘Moderate confidence’). This highlights a distinct behavioral difference in how Grok handles metacognitive prompts compared to the other evaluated models. Although this self-reported confidence does not necessarily correlate with objective clinical accuracy, the ability of an AI model to explicitly express uncertainty or grade its own confidence is highly relevant in a clinical setting. Models that transparently communicate their confidence levels may foster a safer user experience, preventing unwarranted trust in edge-case scenarios—a feature that warrants dedicated investigation in future AI benchmarking studies.

From a clinical perspective, the observed differences between the evaluated LLMs were generally acceptable, suggesting that these models may currently serve as complementary tools for information retrieval rather than substitutes for professional medical advice. Overall, our findings demonstrate the increasing maturity of modern LLMs in supporting patient education in rheumatology, if the generated information is interpreted within the context of expert clinical judgement. This is consistent with recent findings by Shao et al., who reported high accuracy of LLM-generated responses for gout management based on the American College of Rheumatology (ACR) guidelines [23]. Similar to our results, their study demonstrated that although LLMs generally provide guideline-concordant and clinically reliable information, performance varies across models. Taken together, these findings suggest that LLMs have considerable potential to support patient education across different areas of rheumatology, while emphasizing the continued need for clinician oversight and regular evaluation as these models continue to evolve.

These findings have direct implications for clinical practice and patient safety. The current EULAR recommendations explicitly endorse structured pre-conception and pregnancy counselling for women with RMDs, recognizing the well-documented gap between guideline recommendations and real-world implementation [21]. In settings where access to specialised reproductive rheumatology services is limited, LLMs may help bridge this information gap by providing patients with accessible, guideline-based information. However, they should be considered an adjunct to, rather than a replacement for, individualized counselling by experienced rheumatologists.

A systematic review of LLM applications in patient care confirmed that, while these tools can improve health literacy and support patient communication, significant gaps remain in the standardisation and clinical validation of their outputs across medical specialties [4]. While LLMs may serve a complementary role in preparing patients for clinical consultations, our data indicate that their current performance is insufficient to substitute for expert rheumatological counselling. Comparative analyses of LLM responses to guideline-based clinical questions have consistently concluded that these models may serve as supplementary tools for education and reference, but cannot replace evidence-based clinical judgement, underscoring the need for supervised integration and ongoing validation [10]. Future development of domain-specific, guideline-anchored LLM applications — ideally validated against established clinical standards such as the EULAR recommendations — will be essential to harness the potential of these tools while safeguarding patient safety in this vulnerable population.

Overall, the findings of the analysis contribute to research, and the differences in rheumatologist assessments highlight the need to apply standardized assessment frameworks in future research. In this context, a major methodological strength of our study is the development of the 54-question evaluation framework (Supplementary Table S-1). Formulated through a rigorous expert consensus process and strictly anchored to the latest EULAR recommendations, this comprehensive question set covers 11 core sub-groups of reproductive rheumatology. We propose that this carefully curated framework can serve as a robust, standardized benchmarking tool for future studies. As AI technology rapidly evolves, researchers can utilize this exact question set to longitudinally assess, compare, and validate the clinical performance of newly released LLM versions in this highly specialized domain.

### Limitations

The present study has certain limitations regarding its design and the interpretation of its findings, which should be acknowledged:

- *Temporal variability *Each question was asked only once at a time to avoid variation in responses. This means that the study does not capture the possible variation in the models’ responses if the same questions were asked multiple times or at different times. The performance observed in this study reflects the specific versions of LLMs that were available at the time of the research. As is well known, LLMs undergo frequent updates and iterations. Rapid developments in education and the improvement of LLMs lead to improved performance of newer versions. Consequently, developments in future versions of the models may yield different results, more reliable and accurate than those obtained from the present study. As LLMs evolve rapidly, the versions evaluated in this study may become outdated over time.
- *Subjective assessment* The assessment was conducted by a specific panel of ten rheumatology experts, introducing inherent subjectivity in the assessment. Although the participation of 10 independent reviewers and the use of mean scores helped to mitigate individual bias, the assessments remain inherently linked to subjective factors, particularly the clinical experience, interpretation of guidelines, and professional judgment of the participating experts.
- *Distributional characteristics* The low agreement coefficients observed may be partly due to an asymmetric distribution of expert ratings, a common phenomenon in subjective medical assessments.
- *Prompting strategy* All 54 clinical questions were presented to the LLMs within a single prompt and continuous session. While this ensured a uniform context across models, it may have introduced “prompt fatigue” or challenged the context window limitations of the LLMs. Consequently, the depth or completeness of the generated responses might have been slightly compromised. Future benchmarking studies should consider evaluating models using isolated, multiple sessions (e.g., one prompt per question or per category) to determine if response quality and completeness further improve when the models’ computational attention is not divided across multiple queries.
- *Another limitation* is that only four LLMs were evaluated. Given the rapidly expanding number of available models, our findings cannot be generalized to all current or future LLMs.
- An *additional methodological limitation* is the lack of blinding during the evaluation process. The expert raters were aware of which LLM generated each response. As highlighted by principles for rigorous AI research reporting, this non-blinded approach introduces the risk of brand bias, where raters’ preconceptions about specific AI models (e.g., ChatGPT vs. Grok) might have unconsciously influenced their Likert scores. Future studies should implement strict blinding protocols to ensure completely objective evaluations.

## Conclusions

Our study demonstrates that the four LLMs showed satisfactory and promising performance in generating answers to questions regarding the interaction of family planning, pregnancy and lactation in iRMD. Based on the statistical indicators calculated, the average of the scores of the rheumatologists gave very good results, regarding the three characteristics on which the four models were evaluated. This analysis explains that LLMs have the potential to function as useful “digital assistants” in rheumatology, especially regarding the management of family planning, pregnancy and lactation in women with iRMD. While all evaluated models exhibited similarly high and satisfactory levels of clinical safety, significant differences were observed in their completeness. Specifically, DeepSeek and ChatGPT stand out as the most accurate and comprehensive models, while the responses from Gemini and Grok showed greater variability and lower completeness scores.

Despite the promising prospects for the use of AI in medicine, and in particular, in rheumatology, the management of iRMD during pregnancy remains an extremely complex endeavor that requires personalized, multidisciplinary care. Because the responses generated by LLMs may be characterized by certain ambiguities, resulting in different interpretations by experts, the use of LLMs as stand-alone decision-making aids in real-world clinical practice is currently not fully reliable. Continuous evaluation, increased transparency, and human oversight by experts remain necessary to ensure their safe application in healthcare.

In conclusion, future research should focus on longitudinal evaluations and a broader range of pregnancy-related clinical scenarios to better define the role of LLMs in counselling women with inflammatory rheumatic and musculoskeletal diseases. Given the variability in model responses and their interpretation, standardized evaluation frameworks are needed to assess clinical accuracy, safety, and guideline adherence across successive model versions. While LLMs cannot replace specialist counselling, they may serve as a valuable adjunct by improving access to evidence-based information for women seeking guidance on family planning, pregnancy, and lactation, particularly in settings where specialized rheumatology pregnancy services are not readily available. With appropriate safeguards and ongoing validation, LLMs have the potential to support patient education and shared decision-making in this important area of rheumatology care.

## Supplementary Information

Below is the link to the electronic supplementary material.

Supplementary Material 1

## Data Availability

The data are available from the corresponding author upon reasonable request.
